# *Mycobacterium interjectum* Lung Infection

**DOI:** 10.1155/2013/193830

**Published:** 2013-09-30

**Authors:** M. C. Mirant-Borde, S. Alvarez, M. M. Johnson

**Affiliations:** ^1^Division of Pulmonary Medicine, Mayo Clinic Florida, 4500 San Pablo Road, Jacksonville, FL 32224, USA; ^2^Division of Pulmonary Medicine and Infectious Disease, Mayo Clinic Florida, USA

## Abstract

A 62-year-old male presented with productive cough, weight loss, and night sweats. CXR revealed a right upper lobe cavitary lesion. Evaluation was negative for *Mycobacterium tuberculosis*, and sputum revealed *Mycobacterium avium intracellulare* (MAI). Since his clinical course was atypical for MAI, further investigations were pursued which identified *Mycobacterium interjectum* in lung specimens, a very rarely described etiology of pulmonary disease. Appropriate therapy with rifampin, intravenous amikacin, trimethoprim/sulfamethoxazole (TMP/SMX), and ethambutol resulted in clinical and radiographic improvement. This is the third case described over a period of 20 years of destructive lung disease in an immunocompetent adult due to *M. interjectum*.

## 1. Introduction

Various species of *Mycobacterium* lead to human lung disease. *M. tuberculosis* has historically been the most common mycobacterial pulmonary pathogen, but numerous nontuberculous mycobacteria (NTM) are increasingly identified as a cause of pulmonary infections. 

## 2. Case Description

**Figure 1:**
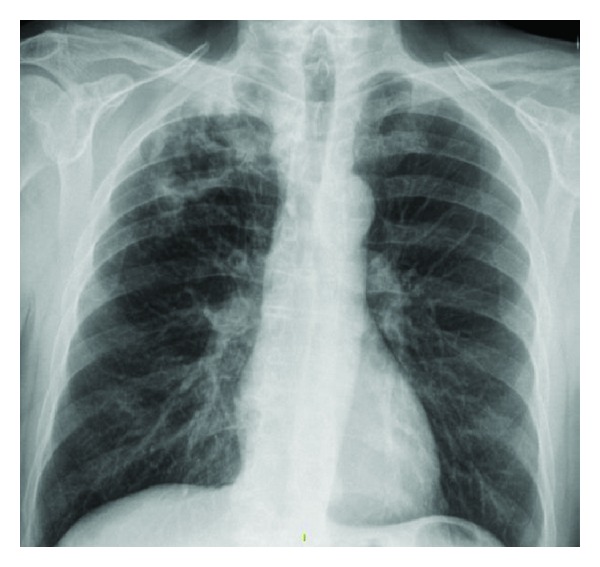
Chest X-ray at presentation.

**Figure 2:**
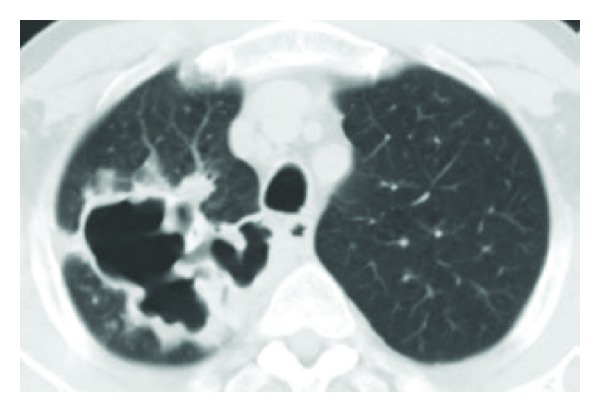
Computed tomography of the chest at presentation.

A 64-year-old male presented to his local physician with drenching night sweats, sixteen-pound unintentional weight loss, and cough productive of dark green sputum for two months. Pertinent negatives included the absence of fever, hemoptysis or dyspnea. Initial chest X-ray (CXR) (Figure 1) demonstrated a right upper lobe cavitary lesion. A 10-day course of ciprofloxacin was provided for a provisional diagnosis of pneumonia without improvement of symptoms. CXR demonstrated progressive cavitation leading to a computed tomography (CT) of the chest (Figure 2) which revealed an 8.6 × 5.9 cm cavity with thick, irregular borders in the right upper lobe with tree-in-bud opacities scattered through the remaining right lung. A sputum sample was negative for *M. tuberculosis* by RNA amplification analysis, and Quantiferon-Gold test was negative. Sputum samples ultimately grew *Mycobacterium avium intracellulare* (MAI) prompting treatment with azithromycin, ethambutol, and rifampin which was started almost five months after the onset of symptoms. He presented to our clinic for a second opinion. Further history was notable for current tobacco abuse of 40 pack-year duration and no obvious risk factors for HIV infection. He reported that one of his coworkers was recently placed on isolation for presumed tuberculosis, but this diagnosis was subsequently excluded. He denied any other exposure to tuberculosis or recent travel. Upon presentation, the patient looked well, without respiratory distress, and was afebrile. Physical examination was unremarkable.

Pertinent laboratory testing revealed normal complete blood count, renal and hepatic panel, negative HIV serology, and normal CD4 helper cells and immunoglobulin levels. CD8 lymphocytes were slightly decreased to 169/*μ*L (normal values were between 180 and 1170/*μ*L). 

A bronchoscopy with bronchoalveolar lavage (BAL) was performed because his clinical course was atypical for MAI infection, and he had not responded to therapy. *Scopulariopsis* spp. and acid fast bacilli were present on direct examination of smears, but RNA amplification was negative for *M. tuberculosis*. No MAI was isolated. 

Based on the initial BAL results, voriconazole, was added and azithromycin was replaced by clarithromycin. Repeated chest CT scans showed disease progression with new involvement of the left lung.

 Ultimately, *Mycobacterium interjectum *was identified in the BAL cultures through DNA sequencing 2 months after presentation to our clinic, and levofloxacin was added to his antibiotic regimen. Susceptibility testing showed sensitivity to rifampin (MIC < 0.5 mcg/mL), rifabutin (MIC < 0.12 mcg/mL), ethambutol (MIC = 4 mcg/mL), clarithromycin (MIC < 4 mcg/mL), streptomycin, and clofazimine and resistance to ciprofloxacin. Rifampin and ethambutol demonstrated synergy. Further susceptibility testing differed in that the isolate was resistant to ethambutol and sensitive to Trimethoprim/sulfamethoxazole (TMP/SMX) and amikacin. Thus, treatment was changed to rifampin, intravenous amikacin, TMP/SMX, and voriconazole. Ethambutol was discontinued but subsequently restarted by his local physician in view of the discrepant results in susceptibility testing between the 2 laboratories. Voriconazole was later discontinued due to its interaction with rifampin and the impression that *Scopulariopsis* most likely represented a saprophyte or contaminant. Amikacin was discontinued after 2 months in face of radiologic and clinical improvement. 

Following antimicrobial therapy, night sweats and weight loss resolved, and cough and sputum production markedly decreased. Chest CT scan performed after six months of current therapy showed stability of the right apical cavity and substantial improvements in the remaining parenchymal disease. Sputum was negative for acid fast bacilli and fungus at 6 months. The patient has continued to improve clinically and is planned to continue therapy with rifampin, ethambutol, clarithromycin, and TMP/SMX for one year after the first negative sputum culture for mycobacteria. He has tolerated drug therapy well with no recognized complications of treatment. The patient declined consideration of surgical resection of the persistent cavity. 

## 3. Discussion

*M. interjectum* is a rare and newly described cause of human infection. It was first described in 1993, causing cervical lymphadenitis in an 18-month-old German boy [1]. It was subsequently reported in 9 pediatric cases of necrotizing lymphadenitis [2–6]. It has also been isolated in the sputum of patients with chronic obstructive lung disease or HIV infection, in the urine of an asymptomatic elderly female, and in the stool of an AIDS patient with diarrhea [7, 8]. Fukuoka et al. described a case of an immunosuppressed Japanese woman with polyangiitis who developed multiple cutaneous abscesses infected with *M. interjectum* requiring repeated surgical resection [9]. In one case of a female alcoholic patient with meningoencephalitis, the cerebrospinal fluid culture presented coisolation of *M. malmoense* and *M. interjectum*. Most of the pediatric cases failed antibiotic therapy and required total resection for definite cure. In most cases of isolation of *M. interjectum* in adult patients, the organism was considered clinically insignificant and did not require treatment. 

Only 2 cases of cavitary lung disease in immunocompetent adults have been reliably described. The first was reported in 1994, in nonsmoker Moroccan woman aged 52 years at the onset of symptoms with progressive destructive lung disease. There was repeated isolation of *M. interjectum* over a period of 10 years, which was refractory to several antibiotic regimens [10]. Another case of cavitary lung disease mimicking tuberculosis was described by Lacasa et al. in 2005, in Spain. The patient responded to an initial 6-month empiric treatment with rifampicin, isoniazid, and pyrazinamide. He relapsed after 18 months showing resistance to all of the previously used antibiotics and, again, responded to a regimen of combined clarithromycin, levofloxacin, and streptomycin [11]. Another male patient from El Salvador with stable pulmonary opacities had *M. interjectum* isolated from sputum, but we lack information about his clinical background [12]. 

*M. interjectum* is phylogenetically found between the fast and slow growing nontuberculous mycobacteria (NTM), leading to its nomenclature, and is similar in its phenotypical and biochemical characteristics to *M. scrofulaceum* (except for hydrolysis of urea which is positive in most cases of *M. scrofulaceum*) and *M. gordonae* [12, 13]. It produces inconsistent reactions for pyrazinamidase, urease, and heat stable catalase [12]. Misclassifications have involved both of these organisms, and accurate diagnosis requires sequencing of the 16S rDNA that codes the ribosomal RNA or high-performance liquid chromatography (HPLC) of the mycolic acids of the cell wall, a more time-consuming and cumbersome technique [2]. Although commonly there is agreement between these tests, discrepant results have been reported [12, 14], and it appears that variants within the *M. interjectum* species may express different HPLC patterns [12]. However, closely related mycobacteria may not be differentiated by 16S rDNA locus polymorphisms, and an algorithm presented by Harmsen et al. is available for this purpose [15]. A newly developed technique that analyzes variations of the heat shock protein (hsp65) gene by PCR restriction fragment polymorphisms has recently described the patterns of rarely isolated mycobacteria, including a new species of *M. interjectum* [16]. Lack of standardization and incomplete pattern libraries may prove to be problematic. In many cases, combination of techniques may be necessary for correct identification of the mycobacterial species [16]. 

Routes of transmission and susceptibility to pulmonary infection from *M. interjectum* are incompletely understood. It is hypothesized that unrecognized immune abnormalities or structural lung disease may predispose to disease. It is known that tumor necrosis factor (TNF) and interferon (INF) are key regulators of mycobacterial defense [17]. Animal data supports a role for deficiencies in INF *γ* monocyte release in *M*. *interjectum* infection [13]. 

Susceptibility testing has demonstrated multidrug resistance, including resistance to one or more of the following: isoniazid, para-aminosalicylic acid (PAS), pyrazinamide, streptomycin, fluoroquinolones, clarithromycin, and rifampin. Primary isoniazid resistance appears to be commonly encountered. The reported variability in drug sensitivity mandates susceptibility testing when this agent is identified as a human pathogen. 

To the best of our knowledge, our case is the third reliably described of destructive lung disease in an immunocompetent adult due to *M. interjectum* since the original report 20 years earlier. We believe the coisolation of *Scopulariopsis* represented saprophytic growth or contaminant. The patient's clinical course improved, and subsequent sputum cultures were negative for fungi, despite only receiving a brief course of voriconazole, further supporting this hypothesis. 

An ever increasing spectrum of pathogenic NTM is recognized to cause human lung infection. The clinical manifestations vary from indolent to very aggressive. Clinicians must be aware of the difficulties in accurately identifying distinct NTM species as misidentification may lead to inappropriate therapy. Although the advent of 16s rDNA sequencing, a more rapid and reliable technique for NTM identification, will likely decrease misclassification, failure to improve on therapy should prompt reexamination. 

This case describes cavitary lung disease due to *M. interjectum*, a rare NTM species causing lung disease. It is possible that many cases of *M. interjectum* producing destructive lung disease have been misdiagnosed in the past and that the prevalence of disease is higher than previously suspected. Outgrowth by other NTM could have also contributed to underdiagnosis, a problem that is overcome by DNA sequencing [2]. Due to phenotypic similarities with other NTM but different drug susceptibility patterns, accurate identification of this organism is necessary. More importantly, this case illustrates the need for repeat investigations in the absence of clinical improvement on presumed appropriate therapy.

## References

[1] Springer B, Kirschner P, Rost-Meyer G, Schröder KH, Kroppenstedt RM, Böttger EC (1993). Mycobacterium interjectum, a new species isolated from a patient with chronic lymphadenitis. *Journal of Clinical Microbiology*.

[2] Tuerlinckx D, Fauville-Dufaux M, Bodart E, Bogaerts P, Dupont B, Glupczynski Y (2009). Submandibular lymphadenitis caused by Mycobacterium interjectum: contribution of new diagnostic tools. *European Journal of Pediatrics*.

[3] de Baere T, Moerman M, Rigouts L (2001). Mycobacterium interjectum as causative agent of cervical lymphadenitis. *Journal of Clinical Microbiology*.

[4] Remacha MA, Esteban A, Parra MI, Jiménez MS (2007). Case report—cervical lymphadenitis due to mycobacterium interjectum. *Pediatric Pulmonology*.

[5] Rose M, Kitz R, Mischke A, Enzensberger R, Schneider V, Zielen S (2004). Lymphadenitis cervicalis due to Mycobacterium interjectum in immunocompetent children. *Acta Paediatrica*.

[6] Rustscheff S, Maroti L, Holberg-Petersen M, Steinbakk M, Hoffner SE (2000). Mycobacterium interjectum: a new pathogen in humans?. *Scandinavian Journal of Infectious Diseases*.

[7] Green BA, Afessa B (2000). Isolation of Mycobacterium interjectum in an AIDS patient with diarrhea. *AIDS*.

[8] Tortoli E, Kirschner P, Bartoloni A (1996). Isolation of an unusual mycobacterium from an AIDS patient. *Journal of Clinical Microbiology*.

[9] Fukuoka M, Matsumura Y, Kore-Eda S, Iinuma Y, Miyachi Y (2008). Cutaneous infection due to Mycobacterium interjectum in an immunosuppressed patient with microscopic polyangiitis. *The British Journal of Dermatology*.

[10] Emler S, Rochat T, Rohner P (1994). Chronic destructive lung disease associated with a novel mycobacterium. *American Journal of Respiratory and Critical Care Medicine*.

[11] Lacasa JM, Cuchi E, Font R (2009). Mycobacterium interjectum as a cause of lung disease mimicking tuberculosis. *International Journal of Tuberculosis and Lung Disease*.

[12] Lumb R, Goodwin A, Ratcliff R, Stapledon R, Holland A, Bastian I (1997). Phenotypic and molecular characterization of three clinical isolates of Mycobacterium interjectum. *Journal of Clinical Microbiology*.

[13] Ehlers S, Richter E (2001). Differential requirement for interferon-*γ* to restrict the growth of or eliminate some recently identified species of nontuberculous mycobacteria in vivo. *Clinical and Experimental Immunology*.

[14] Tortoli E, Bartoloni A, Burrini C (1996). Characterization of an isolate of the newly described species Mycobacterium interjectum. *Zentralblatt für Bakteriologie*.

[15] Harmsen D, Dostal S, Roth A (2003). RIDOM: comprehensive and public sequence database for identification of Mycobacterium species. *BMC Infectious Diseases*.

[16] Häfner B, Haag H, Geiss H-K, Nolte O (2004). Different molecular methods for the identification of rarely isolated non-tuberculous mycobacteria and description of new hsp65 restriction fragment length polymorphism patterns. *Molecular and Cellular Probes*.

[17] Holland SM (2001). Immunotherapy of mycobacterial infections. *Seminars in Respiratory Infections*.

